# Personal

**Published:** 1898-04

**Authors:** 


### Personal.

Dr. Edward N. Brush, of Baltimore, superintendent of the Sheppard and Enoch Pratt Hospital, gave an elaborate dinner about the middle of March at his hospital in honor of the twentieth anniversary of the date of his connection with hospitals for the insane. Many alienists and neurologists were present. Dr. Brush has been most successful in his work at the institutions with which he has been connected, and especially is his administration of the Sheppard and Enoch Pratt Hospital to be commended. Dr. Brush was formerly a resident of Buffalo and an associate editor of this journal.

---

Dr. William House (Univ. of Buffalo, '95,) has located in Buffalo at The Wayne, 1108 Main street. Dr. House served fifteen months as interne at the Erie county hospital after graduation and eighteen months as resident physician at the Manhattan state hospital. His hours are 8 to 10 in the morning, 1.30 to 3 in the afternoon and 7 to 8 in the evening.

---

Dr. Dawson Williams has been appointed editor of the *British Medical Journal*, vice Ernest Hart, deceased. Dr. Williams has been connected with the *Journal* for seventeen years and for part of the time as assistant editor. Mr. C. Lewis Taylor, who has been sub-editor for the last eleven years, becomes assistant editor in the place of Dr. Williams.

---

Dr. Thomas Lothrop, of 153 Delaware avenue, Buffalo, who has been away for the benefit of his health, has returned very much improved and we trust will be soon quite well again. Dr. Lothrop is one of the hard workers in the profession, one of its most useful members and a co-editor of this Journal.

---

Dr. Frank Whitehill Hinkel, of Buffalo, has removed his office from 305 Delaware avenue to 412 Franklin street. This is a most convenient location and is accessible by street cars from all sections of the city.

Dr. Marcel Hartwig, of Buffalo, has removed from 34 E. Huron street to 884 Main street. This is a most admirable location for a physician and Dr. Hartwig is to be congratulated upon securing it.

